# Integrated virtual and cadaveric dissection laboratories enhance first year medical students’ anatomy experience: a pilot study

**DOI:** 10.1186/s12909-019-1806-5

**Published:** 2019-10-07

**Authors:** Kathryn E. Darras, Rebecca Spouge, Rose Hatala, Savvas Nicolaou, Jeff Hu, Anne Worthington, Claudia Krebs, Bruce B. Forster

**Affiliations:** 1 Department of Radiology, Faculty of Medicine, University of British Columbia, 11th Floor, 2775 Laurel St, Vancouver, BC V5Z 1M9 Canada; 2 Department of Medicine, Faculty of Medicine, University of British Columbia, Vancouver, Canada; 3 Evaluation Studies Unit, Faculty of Medicine, University of British Columbia, Vancouver, Canada; 4 Department of Cellular and Physiologic Sciences, Faculty of Medicine, University of British Columbia, Vancouver, Canada

**Keywords:** Education technology, Radiology, Medicine, Cadaver, Dissection, 3D image reconstruction, Medical education

## Abstract

**Background:**

Radiology integration into medical anatomy courses is well established, but there is a paucity of literature on integrating virtual dissection into cadaveric dissection laboratories. Virtual dissection is the digital dissection of medical images on touchscreen anatomy visualization tables. The purpose of this pilot study was to investigate the feasibility of integrating virtual dissection into a first-year medical cadaver-based anatomy course and to assess students’ overall attitude towards this new technology.

**Methods:**

All students in first-year medicine at a single medical school participated in this study (*n* = 292). Six virtual dissection laboratories, which focused on normal anatomy, were developed and integrated into a cadaver-based anatomy course. The virtual dissection table (VDT) was also integrated into the final anatomy spot exam. Following the course, students completed a short evidence-informed survey which was developed using a theoretical framework for curriculum evaluation. Numerical data were tabulated, and qualitative content analysis was performed on students’ unstructured comments.

**Results:**

The survey response rate was 69.2% (*n* = 202/292). Most (78.7%) students reported that virtual dissection enhanced their understanding of the cadaveric anatomy and the clinical applications of anatomy. Most (73.8%) students also felt that the VDT was an effective use of the laboratory time. Thirteen narrative comments were collected, most of which (61.5%) identified strengths of the curriculum.

**Conclusions:**

In this pilot study, students perceived that their learning was enhanced when virtual dissection was combined with a cadaver-based anatomy laboratory. This study demonstrates that there is potential for virtual dissection to augment cadaveric dissection in medical education.

## Background

Anatomy is a fundamental component of medical science, providing students with the knowledge to understand the physical exam, subsequent medical investigations, and how disease affects the human body [1, 2]. In many curricula there has been a trend away from full body cadaveric dissection due to increased curricular emphasis on early clinical exposure, the increasing costs of cadaveric laboratories, and the decreased curricular time due to the inclusion of new subjects in medical school [3, 4]. As a result, educational technologies have been used in the classroom to make the most efficient use of curricular time. Educators need to find new ways to teach anatomy in a way that is clinically relevant and sustainable [5, 6].

Radiology (i.e. medical imaging) is used every day to show anatomy to surgeons and other physicians caring for patients. It is now expected that graduating medical students have a basic understanding of interpreting imaging studies [7]. Consequently, radiology is being increasingly integrated into medical anatomy courses with numerous studies documenting successful integration [8–12]. As a technology-driven speciality, radiology is evolving rapidly and now it is possible to incorporate digital three-dimensional (3D) patient images into curricula rather than two dimensional (2D) images. Of the radiology imaging modalities, the most powerful is computed tomography (CT) which aquires volumetric images that encode the anatomy by density. CT scans are viewed in 2D and 3D daily by physicians in their care of patients. 3D reconstructions of CT scans are becoming more common in anatomy education, especially with recent interest in 3D printing as a teaching tool.

Virtual dissection is an emerging way to interact with 3D reconstructions which can be used in medical anatomy education [13, 14]. During virtual dissection, medical imaging datasets are loaded into a virtual dissection table (VDT), viewed in 3D, and students are able to manipulate the images using digital tools to perform their dissection [15]. They can dissect through skin, muscles and bones of their patient and view the anatomy in a living person. Radiology images themselves do not constitute virtual dissection; students must be able to interact with the data to dissect away tissue layers similar to a cadaver on human virtual cadavers in order for this term to be applied. To date, there has only been one study demonstrating the added value of virtual dissection with CT scans in human anatomy teaching and a few studies showing the educational benefits of incorporating 3D technologies into human anatomy teaching [16]. In this study, the incorporation of cadaver CT scans into VDTs significantly improved medical students’ performance in anatomy [14]. CT scans of living patients have many advantages to CT scans of cadavers. For example, students can see “living anatomy” like blood in the vessels and air in the lungs as well as active disease processes, such as inflammation. Most importantly, physicians look at CT scans of living patients in clinical practice, and exposing medical students to these images improves the authenticity of their learning experience.

The purpose of this pilot study was to integrate virtual dissection using CT scans from living patients into first-year cadaveric dissection laboratories and to assess students’ reaction towards this new technology.

## Methods

### Participants

All students in the first-year of a four-year undergraduate medical program at a single institution participated in this study (*n* = 292). Institutional review board approval (and therefore participant consent) was not required according to national regulations (TCPS2 2014, Article 2.5) [17].

### Materials

#### Integrated cadaver and virtual dissection laboratory development

The first semester includes 12 Cadaveric Dissection Labs, which cover the spine, thorax, abdomen, and pelvis. The anatomy learning objectives from these labs were reviewed and those objectives deemed relevant to radiology and consistent with established standards for medical student radiology teaching were adapted for the Virtual Dissection Labs [18]. In total, there were six virtual dissection labs developed: spine, lungs and mediastinum, heart, abdominal vasculature, abdominal organs, and bony pelvis. Each virtual dissection lab was developed so that it complimented the content presented in the cadaveric dissection lab (i.e. Integrated Lab). Patient CT scans were selected from the VDT database although it is also possible for educators to upload CT scans from their own institutions (Sectra AB, Linköping, Sweden) [15]. An example of an integrated cadaveric-virtual dissection lab is shown in Table 1.

**Table 1:** Objectives of the spine integrated cadaveric-virtual dissection laboratory. The virtual dissection component of the labs aimed to show students anatomy that was challenging to expose in the cadaver to provide students with a comprehensive learning experience

	Cadaveric Dissection	Virtual Dissection
Technique	Cadaver prone, laminectomy dissection	Bone windows, whole skeleton included on single CT image
Main objectives	● Identify the key bony and ligamentous landmarks of the spine ● Describe how the anatomy of the spine contributes to its biomechanics	
Detailed objectives	● Identify the three meninges ● Identify parts of a typical spinal nerve ● Describe how a spinal nerve exits the vertebral column ● Describe how cranial and spinal nerves are named	● Identify the bony anatomy of the craniocervical junction ● Describe the relationship between the dens and the skull base ● Describe the unique anatomic features of cervical, thoracic, and lumbar vertebrae

The Integrated Labs ranged from 2 to 3 h in length. Students spent most of this time completing their cadaveric dissection with 10 min allotted to the Virtual Dissection component. Students rotated to the VDT in groups of 10–15 where a radiologist demonstrated the normal anatomy on CT scans through virtual dissection. This split was determined by the number of students and number of VDTs in the lab. Due to resource and time constraints, students did not perform their own virtual dissections.

In addition, the VDT was included in the final anatomy exam as one of the practical spot exam questions, primarily to demonstrate feasibility of including virtual dissection questions in a standardized practical anatomy exam. For this question, the dens of the cervical spine was labeled on the 3D CT scan with an arrow using the VDT software. The image was then rotated to obscure the arrowhead, forcing students to interact with the VDT in order to identify the labeled structure. After each student completed this question, an exam invigilator reset the image to the original position for the next student.

#### Survey development

The post-course survey was based on the four levels of Kirkpatrick’s Hierarchy for curriculum analysis and evaluation. This model considers the educational value of the curriculum using four levels: (1) Reaction, (2) Learning, (3) Behaviour, and (4) Results. For this pilot project, learners’ reactions (i.e. Level 1) to the integrated curriculum were assessed [19]. The survey consisted of three questions relating to how the students perceived the benefits of this curricular integration. Prior literature has suggested that these are the areas which students can competently evaluate teaching and curriculum [20]. For each item, students were asked their agreement with a statement on a 5-point Likert scale (1 = strongly disagree; 2 = disagree; 3 = neither agree nor disagree; 4 = agree; 5 = strongly agree). In addition, students were asked to provide unstructured comments related to their experience with virtual dissection.

### Procedure

All students in the first-year medical class received the online survey which was sent out one week following the course and remained open for four weeks, with weekly email reminders. The survey was voluntary and anonymous. The survey results were tabulated and summarized to identify trends. Qualitative content analysis was performed on the unstructured comments submitted by students.

## Results

The survey response rate was 69.2% (*n* = 202/292). Most students (78.7%) reported that seeing the radiographic anatomy on the VDT enhanced their understanding of the cadaveric anatomy and that the content shown on the VDT enhanced their understanding of the clinical applications of anatomy. Most respondents (73.8%) also felt that the VDT was an effective use of the cadaveric laboratory time. Results are summarized in Table 2.

**Table 2:** Student feedback regarding the Virtual Dissection Labs in the Integrated Lab Curriculum. For each item, students were asked their agreement with the statement on a 5-point Likert scale (1 = strongly disagree; 2 = disagree; 3 = neither agree nor disagree; 4 = agree; 5 = strongly agree). N = number of respondents; SD = standard deviation.

Survey Item	N	Mean	SD
Seeing the radiographic anatomy on the Virtual Dissection Table enhanced my understanding of cadaveric anatomy.	202	4.08	.837
The content shown on the Virtual Dissection Table enhanced my understanding of the clinical applications of anatomy.	202	4.09	.882
Teaching at the Virtual Dissection Table was an effective use of time in the lab.	201	3.97	.908

Thirteen individual comments were submitted by 13 different students regarding their experience in the integrated cadaveric and virtual dissection labs. Eight (61.5%) of these comments identified strengths of the curriculum and 5 (38.4%) of these comments identified weaknesses. From the 8 positive comments, the most commonly cited strength of the curriculum was the ability to see the same anatomic structures on two different modalities (Table 3). Several students also described the ability to see 3D anatomic images as a major advantage of virtual dissection. The five comments that cited a weakness all described the same issue; students felt that they did not have sufficient “hands-on” practice in performing virtual.

**Table 3:** Select student comments regarding the strengths of the Integrated Lab Curriculum

Respondent	Comment
A	“[Virtual dissection] was so valuable for our learning. I was able to gain a better understanding of anatomy being able to see all the structures in 3D.”
B	“[Virtual dissection] made it a lot easier to understand the anatomy and CT scans because it could be so easily manipulated.”
C	“Having a variety of ways to learn the material helped me to think about it from different perspectives and viewpoints.”

## Discussion

Our pilot study demonstrates a novel way to structure a medical student anatomy laboratory, incorporating both virtual and cadaveric dissection, and to introduce students to basic radiology concepts. Although there are many studies on the use of radiology images in anatomy teaching, there is a paucity of literature on the use of VDTs to augment medical students’ cadaveric dissection experience. A recent study by Paech et al. used this technology to perform simultaneous virtual and cadaveric dissection using CT scans of the cadavers and not of real-life patient cases [14]. Cadaver CT scans may be inferior for teaching anatomy because the do not demonstrate living anatomy, which is essential for physicians to understand. For example, it is important to be able to recognize a normal bowel gas pattern on radiological studies in order to diagnose certain diseases, such as a bowel obstruction. Students would not be able to learn the normal bowel gas pattern on a cadaver CT scan because the scan is of a deceased individual [21]. One of the strengths of virtual dissection using patient CT scans, is that the scan captures living physiology and anatomy, such as the normally aerated aerodigestive tract.

Overall, students reported a positive learning experience when participating in our integrated virtual-cadaveric dissection curriculum and indicated that this pilot curriculum enhanced their understanding of the cadaveric anatomy and the clinical applications of anatomy. This supports the principle that virtual or digital anatomy experiences should support rather than replace cadaveric dissection [22]. Paech et al. reported that students demonstrated significant improvement in their anatomy knowledge with this integrated pedagogical approach [14]. In addition, a meta-analysis by Yammine and Violato found that 3D visualization technologies showed higher factual knowledge, better results in spatial knowledge and improved student satisfaction and learners’ perception of teaching effectiveness when compared to non-virtual teaching strategies [16]. Further investigation would be required to determine if similar educational benefits are seen in virtual dissection using living patient CT scans.

We found that students reported that virtual dissection improved their understanding of the clinical applications of anatomy. Vertical integration, or the early integration of clinical concepts into pre-clinical medical student education, is an important part of most medical curricula. Several studies demonstrate how vertical integration is facilitated in anatomy education by the integration of radiology [10, 23, 24]. In clinical practice, radiologists and other physicians not only view CT scans daily but also use similar (non-touchscreen) software at their workstations to create 3D CT images. Comfort with virtual dissection techniques may therefore better prepare students for viewing CT imaging studies in the clinical environment although further research would be required.

The format of our Integrated Labs are consistent with student preferences for combined anatomy/radiology education [25]. A study by Murphy et al. determined that students at a single institution benefitted from learning radiology with the anatomy content but preferred that radiology be an adjunct rather than primary method of instruction. This model was utilized in our curriculum where students spent only 10–15% of their time in the lab learning through virtual dissection. However, there were still comments from students that they would have liked to have more exposure to virtual dissection during this time, which may account for why only 73.8% of students felt that the VDT was an effective use of laboratory time. If it is not possible to expand anatomy curricula to include virtual dissection due to time constraints, this tool could also be integrated into other parts of the medical curriculum, such as clinical skills or dedicated radiology sessions. It is important for educators to be aware that, while virtual dissection incorporates radiology images and introduces students to imaging concepts, it does not replace a dedicated radiology curriculum which includes concepts beyond anatomy (e.g. imaging appropriateness, radiation risks).

In addition, our pilot study demonstrates the feasibility of integrating a virtual dissection question into a practical lab spot exam. The VDT software enables educators to label any structure similar the traditional cadaver-based spot exam. Although this is achievable, using the VDT in this capacity is very resource intensive, requiring an individual (e.g. teaching assistant or invigilator) to stand next to the VDT to re-orient the image for each student. A recent study by Ikah et al. demonstrated that among Year 2 medical students anatomy exam questions with a clinical stem showed much better discrimination index than non-clinical items [26]. The use of a clinical question through the VDT could be a means to seamlessly integrate clinical scenarios into an anatomy exam.

As with any educational approach, virtual dissection does have some disadvantages. The main limitation is that dissection software relies on the discrimination between tissue densities on a CT scan. For example, structures with the same density on CT scans (e.g. small and large bowel) cannot be clearly separated by virtual dissection. Based on our experience, it is recommended that the curriculum design team include both radiologists and anatomists to ensure a comprehensive understanding of how each type of dissection can best help to fulfil the learning objectives. Another drawback is patient confidentiality if instructors elected to choose CT cases from their own hospital. The VDT system used in this study removed or obscured anything that could potential identify the patient (e.g. facial features, tattoos, image metadata identifiers) and awareness of this issue is key.

Our study demonstrates one way in which virtual and cadaveric dissection can be combined so that students perceive the integrated experience as valuable to their learning. We found that most respondents felt that the virtual dissection labs enhanced their perceived understanding of cadaveric anatomy. These results may indicate that despite the growing use of educational technology and radiology in medical anatomy education, there appears to still be an important role for cadaveric dissection [27]. Our Integrated Labs were designed to harness the advantages of each approach (i.e. cadaver or radiology) in order to provide students with a comprehensive learning experience. For example, we developed curricular objectives to highlight the strength of each type of dissection; objectives that are better seen on CT scan (e.g. craniocervical junction) were aligned with virtual dissection and those that are better seen in cadavers (e.g. nerves) were aligned with cadaveric dissection. Further research is required to determine if the perceived value of this integrated approach results in a significant change in anatomy knowledge.

The main limitation of this study was that it was a brief intervention, performed at a single institution with a robust cadaveric dissection program and a motivated faculty who were keen to incorporate educational technologies, which facilitated the development of Integrated Labs. Not all institutions offer cadaveric dissection, so the virtual dissection labs developed for this pilot study would have to be adapted for other programs. In addition, data collection methods were limited to a student survey, which captured student perceptions of the benefits of the VDT but did not assess its impact on their knowledge or skills. Despite a good response to the survey, only 6.4% of these students provided qualitative data and it is unclear what motivated student to include narrative comments. Unfortunately, no comparative group was used for testing students’ perception. Finally, Kirkpatrick’s model does not take into account all the variables that affect learning, such as learner motivation [28]. Further research is required to understand how this novel educational technology might best be utilized to improve student learning and enhance physician competency.

## Conclusion

This pilot study demonstrates the feasibility of integrating virtual dissection into first year medical school cadaver labs. Overall, students reported a positive learning experience with virtual dissection, feeling that it was an effective use of their time and improved their understanding of the clinical relevance of anatomy as well as cadaveric anatomy.

## Data Availability

The datasets used during the current study are available from the corresponding author on reasonable request.

## Abbreviations

2D: Two-dimensional
3D: Three-dimensional
CT: Computed tomography
TCPS: Tri-Council Policy Statement
VDT: Virtual dissection table
